# Quantitative measures for the management and comparison of annotated genomes

**DOI:** 10.1186/1471-2105-10-67

**Published:** 2009-02-23

**Authors:** Karen Eilbeck, Barry Moore, Carson Holt, Mark Yandell

**Affiliations:** 1 Department of Human genetics, Eccles Institute of Human Genetics, University of Utah and School of Medicine, Salt Lake City, Utah, USA

## Abstract

**Background:**

The ever-increasing number of sequenced and annotated genomes has made management of their annotations a significant undertaking, especially for large eukaryotic genomes containing many thousands of genes. Typically, changes in gene and transcript numbers are used to summarize changes from release to release, but these measures say nothing about changes to individual annotations, nor do they provide any means to identify annotations in need of manual review.

**Results:**

In response, we have developed a suite of quantitative measures to better characterize changes to a genome's annotations between releases, and to prioritize problematic annotations for manual review. We have applied these measures to the annotations of five eukaryotic genomes over multiple releases – *H. sapiens*, *M. musculus*, *D. melanogaster*, *A. gambiae*, and *C. elegans*.

**Conclusion:**

Our results provide the first detailed, historical overview of how these genomes' annotations have changed over the years, and demonstrate the usefulness of these measures for genome annotation management.

## Background

The number of sequenced and annotated genomes is rapidly increasing. There are currently 925 published genomes and 3185 genome sequencing projects underway [1]. Of those underway, over 900 are eukaryotic, genomes whose large size and intron-containing genes complicate annotation. Even assuming as few as 10,000 genes/genome, these new eukaryotic genomes alone will add more than nine million annotations to GenBank. Tools to manage and analyze these gene annotations are badly needed. Consider too that next-generation sequencing technologies will soon make it possible for individual labs to sequence and annotate genomes, thus the number of gene annotations could well exceed one billion in a few years time.

Gene annotations are not static entities, and how to best mange them is a complex and challenging problem. Gene annotations must be tracked from release to release, and problematic annotations identified, reviewed and modified. By nature this is a comparative process. Standardization of formats and database schemas has helped matters greatly. The Sequence Ontology [2] and GMOD projects [3], for example, provide tools and standards that promote database interoperability. This in turn has made possible common formats for data exchange such as CHADO XML [4] and gff3 [5]. The result has been an ever-proliferating number of groups annotating and redistributing their own annotations, independent of the annotation pipelines used by GenBank. Examples include not only model organism databases such as *C. elegans*, and *D. melanogaster *but also emerging model organisms such as the planarian *S. mediterranea *[6]. The growing numbers of annotation providers – and users – is creating a pressing need for tools and techniques for gene annotation management and analysis.

Today, most annotation management and comparison at the whole-genome scale is restricted to analyses of basic traits – for example differences between releases are usually evaluated in terms of gene and transcript numbers [7]. Though indisputably useful, these simple statistics only tell part of the story. Comparisons of different genomes' annotations also suffer from a paucity of measures, with most studies restricted to analyses of protein alignments [8-10]. Here too, new measures of comparison are needed, measures that move beyond the amino acid sequences and take into account other aspects of the annotations such as similarities in intron-exon structures and patterns of alternative splicing.

Some previous work has been done in this area. The Sequence Ontology project [2], for example, has created a categorization system for alternative splicing that can identify problematic annotations for later manual review. The DEBD [11] and ASTRA [12] projects have also proposed genome-wide categorizations of alternative splicing using graph-based approaches. In principle these classification systems could be used for whole-genome annotation management, but to our knowledge they have not yet been applied for this purpose. Furthermore, useful as qualitative classification systems are, quantitative metrics are also needed – measures akin to the sensitivity, specificity and accuracy metrics used by the gene-prediction community to evaluate gene-finder performance [13]. These measures have seen wide use [14-16]. However, they also have recognized shortcomings. Indeed, the recent eGASP contest concluded with a call for new performance measures for alternative splicing and UTR prediction [16]. Moreover, these measures are designed for evaluating gene-prediction algorithms. The problems faced in annotation management are similar in spirit, but distinct enough to require different measures and software. In response to these issues, we have formulated a set of metrics for annotation comparison.

We introduce two new measures to evaluate changes to annotations across releases: Annotation Turnover, and Annotation Edit Distance. Annotation Turnover tracks the addition and deletion of gene annotations from release to release. We show that tracking annotations in this manner supplements traditional gene and transcript counts, allowing the detection of 'resurrection events' – cases where an annotation is created in one release, later deleted, and then after a lapse of one or more releases a new annotation is created at the old genomic location, with no reference to the previous annotation.

We use a second, complementary, measure, called Annotation Edit Distance (AED) to quantify the changes to individual annotations from release to release. AED is similar to performance measures employed by the gene-prediction community, but takes into account aspects of annotations not well addressed by conventional sensitivity/specificity measures [13] such as alternative splicing. AED complements Annotation Turnover and gene and transcript numbers in that it measures structural changes to an annotation. Two releases can differ dramatically from one another, with every annotation's intron-exon structure having been revised, yet still have identical gene and transcript numbers and no Annotation Turnover; AED provides a means to distinguish between a new release with no changes, and one wherein the intron-exon coordinates alone have been altered. Moreover, it provides a means to quantify the extent of these changes.

We also introduce a new measure for quantifying the complexity of alternative splicing, which we call Splice Complexity. Those in the field of gene annotation often speak of one gene as having a more complex pattern of alternative spicing than another. For example, a gene with 20 transcripts, each with different combinations of exons, is said to be more complex than a gene producing two transcripts that differ from one another by only a few nucleotides at their 5' ends. Splice Complexity provides a means to quantify transcriptional complexity; moreover, because it is independent of sequence homology, Splice Complexity can be used to compare any alternatively spliced gene to any other. This makes possible novel, global comparisons of alternate splicing across genomes. We have used Splice Complexity in conjunction with a classification scheme for alternatively spliced genes developed by the Sequence Ontology project [2] in order to obtain a global perspective on alternative splicing in different genomes. These novel analyses suggest that the complexity and mode of alternative splicing varies considerably amongst the different genomes in our collection.

In total we have analyzed over 500,000 annotations in this study. To our knowledge this is the largest meta-analysis of gene annotations ever undertaken. Our results reveal both global differences among the annotations of different genomes and unexpected similarities – demonstrating the utility of these new measures for whole-genome annotation management and for comparative genomics studies.

## Results

Our analyses fall into two classes – *intra*-genome comparisons of annotations that track and summarize genome-wide changes in annotations from release to release, and *inter*-genome comparisons that compare and contrast the annotations of different genomes to one another. We chose five annotated genomes for these analyses: *Homo sapiens*, *Mus musculus*, *Drosophila melanogaster*, *Anopheles gambiae*, and *Caenorhabditis elegans*. For *D. melanogaster *and *C. elegans *we used gff3 [5] releases from FlyBase [17] and WormBase [18] respectively. For *H. sapiens*, *Mus. musculus *and *A. gambiae *we used GenBank releases [19]. We also took practical issues into account when choosing which releases to analyze, such as completeness, and usability. The early gff3 releases of FlyBase and WormBase, for example, were alpha releases designed to troubleshoot the release process; in some cases this precluded effective analyses of some aspects of their contents. In total we analyzed six human GenBank releases (33–36.2), five *M. musculus *GenBank releases (30–36.1), four *D. melanogaster *FlyBase releases (3.2.2-5.1), and five *C. elegans *WormBase releases (WS100-WS176). We also included an *A. gambiae *release (08/2007) from GenBank in some of our analyses. See Additional File 1 for details of the dataset.

### Annotation Edit Distance

**Figure 1:**
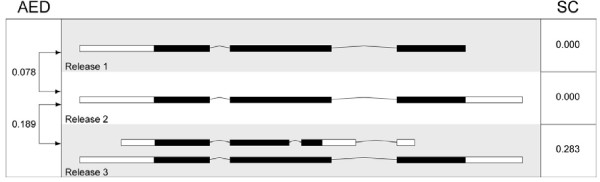
**Annotation Edit Distance and Splice Complexity**. This Figure shows three versions of the same annotation with their corresponding Annotation Edit Distance (AED) and Splice Complexity (SC) values. The left column shows the AED values between the three releases. The right column, SC values for each version. In release 1, the annotation consists of a single transcript with three exons. In release 2, 3' UTR has been added, increasing the AED, but leaving SC unchanged. In release 3, a second, alternatively-spliced transcript has been added, increasing AED and SC. The black portions of each transcript denote its translated portion. See Methods section for the details of the calculation.

**Figure 2:**
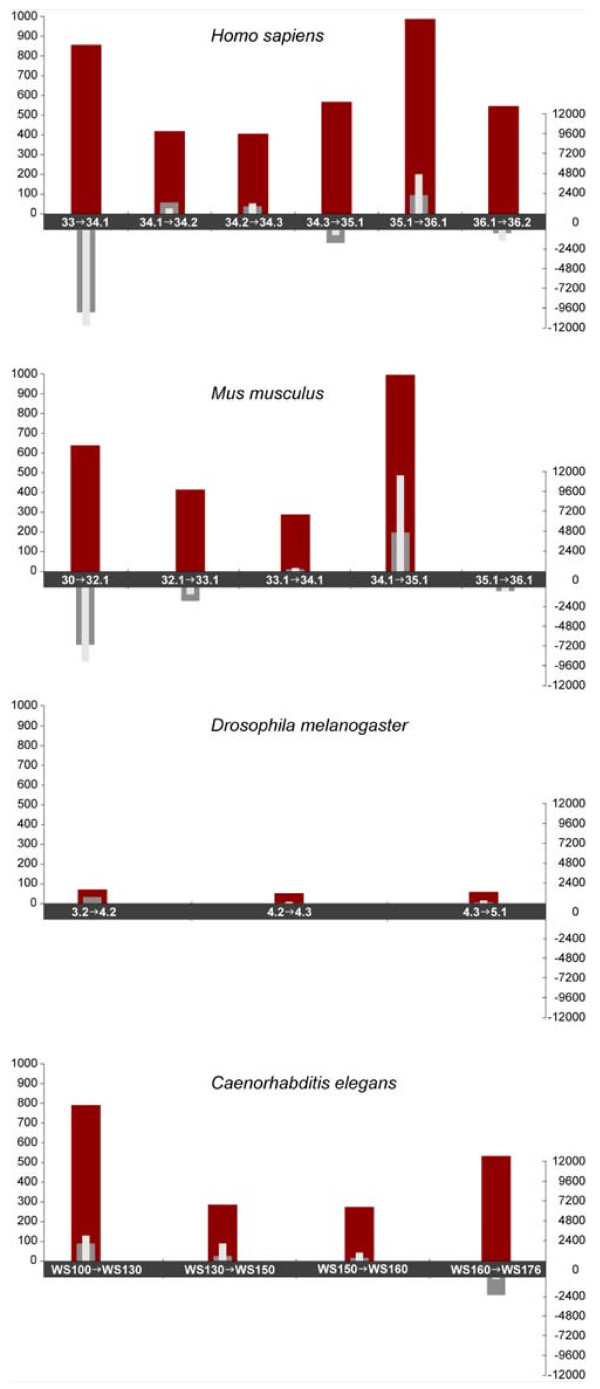
**Cumulative Annotation Edit Distances by release for four genomes**. Pairs of releases are labeled on the *x*-axis. *y*-axis (left-hand side): total AED between the two releases; *y*-axis (right-hand side): total change in gene and transcript numbers between the two releases. red bar: total AED; dark-grey bar: change in gene number between releases; light-grey bar: change in transcript numbers between releases.

We used a measure we term Annotation Edit Distance (AED) to quantify the amount of change to individual annotations between releases (see Methods for details; and Figure 1 for examples). In order to measure rates of annotation revision independently of changes to the underlying assembly, we excluded from these calculations any annotation version-pair with changes to the underlying genomic sequence (see Methods, section entitled Assembly-induced changes). Figure 2 summarizes the total amount of annotation revision between releases for four of the genomes in our dataset. *D. melanogaster *is by far the most stable genome. Though small numbers of new gene annotations have been added incrementally since release 3.2 (10/2004), the vast majority of its annotations have remained unchanged at the level of their transcript coordinates (Figure 2). Overall, 94% the genes in the current release (5.1) have remained unaltered since 2004, and only 0.3% have been altered more than once (TABLES 1 &2). The *C. elegans *genome, by comparison has undergone significant revision with each release. Although gene and transcript numbers have changed by less than 3% since 2003 (WS100) (Additional File 1), 58% of annotations in the current release have been modified since 2003, 32% more than once (TABLES 1 &2). It is also worth noting that although far fewer *D. melanogaster *annotations than *C. elegans *annotations were revised from release to release, the changes to *D. melanogaster *annotations tended to be of greater in magnitude (TABLE 3); the average AED/modified transcript for *D. melanogaster *was 0.092 compared to 0.058 for *C. elegans*.

**Table 1:** Percentage of Genes in the current release with a history of modification

Organism	AED > 0	Genes	%
*C. elegans*	11,597	20,061	58
*D. melanogaster*	909	14,512	6
*M. musculus*	8,980	31,037	29
*H. sapiens*	12,790	23,342	55

Columns: organism, number of genes modified at least once, number of genes in the most recent release, percentage of genes modified at some point it the past. *C. elegans*: release WS176 (07/2007) annotations since WS100 (05/2003). *D. melanogaster*; release r5.1 (12/2006) annotations since r3.2 (10/2004). *M. musculus*; release 36.1 (5/2006) annotations since 30 (2/2003). *H. sapiens *release 36.2 (9/2006) annotations since 33 (4/2003).

**Table 2:** Percentage of genes in the latest release that have been modified *n *times in their past.

Edits	*C. elegans*	*D. melanogaster*	*M. musculus*	*H. sapiens*
0	40.96	93.40	68.72	36.60
1	26.77	6.29	20.60	30.57
2	19.67	0.29	7.77	16.24
3	10.76	0.01	2.35	11.19
4	1.84		0.55	4.09
5				1.09
6				0.20

The first column gives the number of revisions. Time intervals are the same as those in Table 1. Because not every gene could be identified in every release, percentages do not exactly tally with those in Table 1.

**Table 3:** Average AED per revised transcript.

Organism	Average
*C. elegans*	0.058
*D. melanogaster*	0.092
*H. sapiens*	0.086
*M. musculus*	0.108

Average AED (magnitude of revision) for each transcript in the current release that has been modified at least once in its past. Time intervals are the same as those in Table 1.

*H. sapiens *and *M. musculus *annotations are also undergoing considerable revision from release to release. 55% of current human annotations (release 36.2) have been modified at least once since 2003, with an average AED/revised transcript of 0.086. Substantial numbers of mouse annotations have also undergone revision. 29% of annotations in the release 36.1 (current at time of writing) have been modified at least once since their creation. Finally as Figure 2 makes clear, mouse release 36.1 is somewhat atypical in that no transcript coordinates were altered, though the CDS coordinates of 51 transcripts were changed. In addition, release 36.1 saw the deletion of 487 genes and 501 transcripts (Additional File 1).

These results show how AED naturally supplements gene and transcript numbers. Consideration of gene and transcript numbers alone, for example, would lead one to believe that the *C. elegans *and *D. melanogaster *annotations are both relatively static, when in fact the *C. elegans *annotations are evolving rapidly compared to those of *D. melanogaster*. Considering AED in conjunction with gene and transcript counts also makes it clear that the dynamics of the two invertebrate annotation sets differ markedly from the vertebrate ones, which are characterized by large fluctuations in both gene and transcript numbers – and AED.

### Annotation Turnover

**Figure 3:**
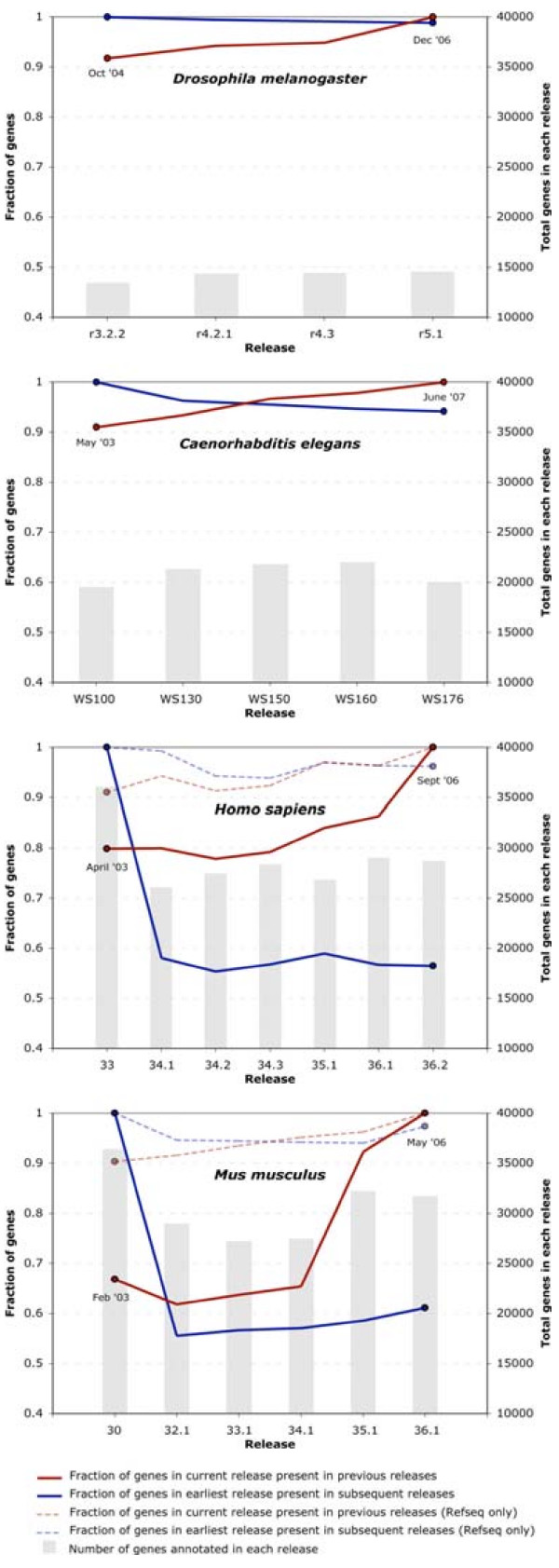
**Annotation Turnover**. Gene Annotations traced from the first release forward (blue line) and from the last release backwards (red line). Dotted lines in the *H. sapiens *and *M. musculus *panels show the same data plotted for RefSeq NM/NR annotations only. *x*-axis: release number. *y*-axis (left-hand side): fraction of genes in the first or last release with reciprocal best hits in subsequent releases. *y*-axis (right-hand side): total number of genes in that release. Release dates for the first and last release surveyed are noted on the Figure.

We also measured annotation turnover – the addition and deletion of gene annotations from release to release (see Methods for details). Figure 3 summarizes annotation turnover from two perspectives: the red line shows the fraction of annotated genes in the current release that were present in prior releases; the blue line, the fraction of annotations in the first release still present in subsequent releases. For example, 92% of the annotations in the latest *D. melanogaster *release were present in release 3.2.2 (10/2004); likewise 99% of annotations present in release 3.2 still exist. These facts together with the low annotation edit distances characteristic of this genome show that since the omnibus release 3 [7,20,21], changes have largely been due to the addition of modest numbers of new genes. The situation is similar for the *C. elegans *genome. It's annotations have undergone a low and balanced rate of annotation turnover: 95% of the WS100 (05/2003) annotations still remained in one form or another as of the WS176 release (06/2007), and 91% of the current annotations were present as long ago as WS100 (05/2003) (Figure 3).

The *H. sapiens *and *M. musculus *genomes have undergone higher rates of annotation turnover than either of the two invertebrate genomes (Figure 3). Less than 60% of annotations present in human release 33 and mouse release 30 were still in existence by the next release, e.g. human release 34.1 and mouse release 32.1. Most of the turnover was due to annotation deletion: between April and October 2003, human gene counts fell by 28% (Additional File 1), and between February and October 2003 mouse gene numbers fell 20%. Since this early clean up, mouse and human gene numbers have risen by 10%. Interestingly, about 1 in 3 (30%) of the new mouse genes are resurrections of release 30 annotations deleted from release 32; this is the underlying cause of the upward trend in blue line since release 32.1 in Figure 3.

We also measured Annotation turnover for the human and mouse Refseq NM and NR annotations [22]. These are shown as dotted lines in the human and mouse panels in Figure 3. Turnover rates for these curated annotations are much lower. For both genomes, over 95% of Refseq NM and NRs present in the first releases in our collection (2003) were still present in last (2006). Likewise, more than 90% of 2006 human and mouse Refseq NM and NRs were present as long ago as 2003. Thus, the NMs and NRs paint a very different picture of annotation turnover, one that closely resembles the *C. elegans *and *D. melanogaster *turnover data (Figure 3), making it clear that most of the turnover in human and mouse genomes has been due to addition and deletion of automatically generated annotations for which there is little experimental support.

### Alternative splicing

Alternatively spliced genes pose special challenges for annotation efforts. Because they are not predicted by most gene finders, and predicted with poor accuracy by those that do [23], alternatively-spliced transcripts are generally the product of manual annotation efforts. As such, they provide an important indication of the extent and completeness of active curation efforts. 15% of human genes (release 36.2), 7% of mouse (release 36.1), 24% of *D. melanogaster*, 9% of mosquito, and 19% of *C. elegans *genes have more than one annotated transcript (Additional File 1).

There has been a strong trend towards ever increasing numbers of alternatively spliced annotations from release to release for every genome in our collection (Additional File 1). Although this trend illustrates the growing focus on the annotation of alternatively spliced genes, it says nothing about how the contents of alternatively spliced annotations have evolved from release to release and how they differ between genomes. We have undertaken two analyses to address these points. First, we classified alternatively spliced annotations using a scheme developed by the Sequence Ontology. We also used a measure we term Splice Complexity (see Methods) to quantify the complexity of each alternatively spliced annotation.

### SO-based classifications

**Figure 4:**
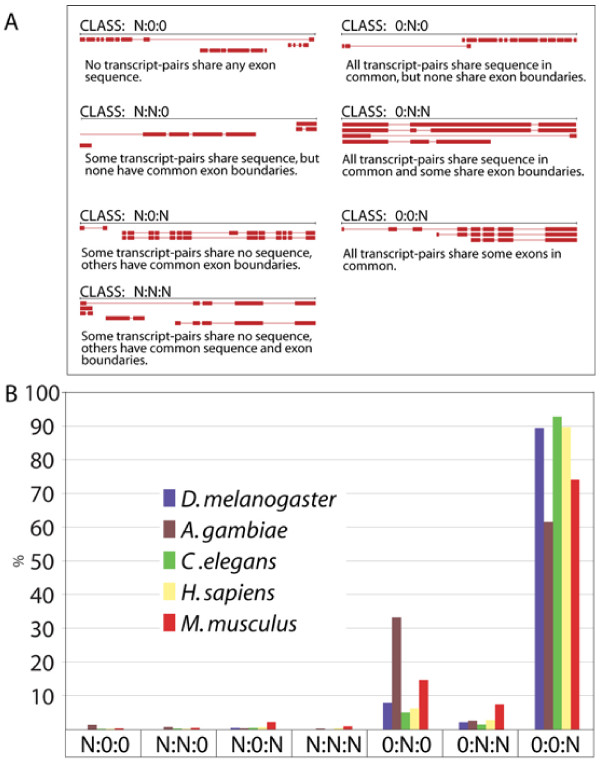
**Sequence Ontology-based classification of alternative spliced annotations in five annotated genomes**. The Sequence Ontology schema classifies alternatively-transcribed and alternatively-spliced genes into seven different classes; this is done by first grouping their transcript-pairs into three classes: (1) pairs of transcripts that share no sequence in common, (2) transcript-pairs with sequence in common, but which share no exon-boundaries precisely in common, and (3) transcript-pairs that share one or more exons in common. This process results in seven classes of gene; N:0:0 genes for example encode only transcripts that do not overlap. **Panel A **provides a key for each of the seven classes, consisting of an alternatively spliced gene exhibiting a representative pattern of alternative splicing and its associated classification, *e.g*. CLASS N:0:0 (Top left). **Panel B **shows the percentage of genes in each genome falling into each class.

We used a classification system developed by the Sequence Ontology group [2] to characterize the alternatively spliced annotations in our collection of annotated genomes; this is the first application of this classification system to multiple genomes and releases. These data are summarized in Figure 4B. The Sequence Ontology's classification system categorizes an alternatively spliced gene into one of seven modes based upon shared and unique exons among its transcripts [2] (see Figure 4A for details). Some classes of alternative splicing are especially indicative of errors in annotation. Class N:0:0 genes, for example, have multiple transcripts that share no exon sequence in common. Thus N:0:0 annotations are likely to be multiple genes, incorrectly merged into a single annotation. When considering N:0:0 annotations it is important to understand that even though when viewed in a genome browser these appear to be separate genes, they are – within the host database – a single gene. Thus queries to the database to determine gene numbers, the number of alternatively spliced transcripts, etc are distorted by these mis-annotations. Also suspect are 0:N:0 genes. None of these genes' transcripts share any exon coordinates precisely in common; hence, each transcript encodes a slightly different peptide. Though a formal possibility, Eilbeck *et al *[2] suggest that 0:N:0 annotations should be subjected to manual review in order to make sure that their unusual patterns of alternative splicing are confirmed by EST evidence.

Previous work on the *D. melanogaster *genome [2] has shown that the vast majority of its alternatively spliced genes belong to the 0:0:N class. We find that this trend also holds true for every genome in our collection (Figure 4B). However, the *M. musculus *and *A. gambiae *genomes are enriched for problematic annotations: 33% of *A. gambiae *and 15% of *M. musculus *alternatively spliced genes, for example, consist of transcripts lacking any exon borders in common (c.f. 0:N:0 class in Figure 4A Error! Reference source not found.). The high percentage of such genes in the *Mus *and *Anopheles *genomes indicates that these annotations are in need of review, as many of them may be mis-annotated. Conventional release statistics such as gene and transcript numbers or percentages of alt-spliced genes can never reveal trends such as these. Thus, these results highlight the usefulness of the SO classification scheme for annotation management.

### Splice complexity

To further characterize alternatively spliced genes, we developed a new measure that we term Splice Complexity. Splice Complexity provides a means to quantify (rather than classify) the complexity of a genome's alternatively spliced annotations; it thus naturally complements existing classification systems such as the Sequence Ontology's [2] and graph-based splicing schemes [11,12]. The Methods Section describes in detail how Splice Complexity is calculated.

**Figure 5:**
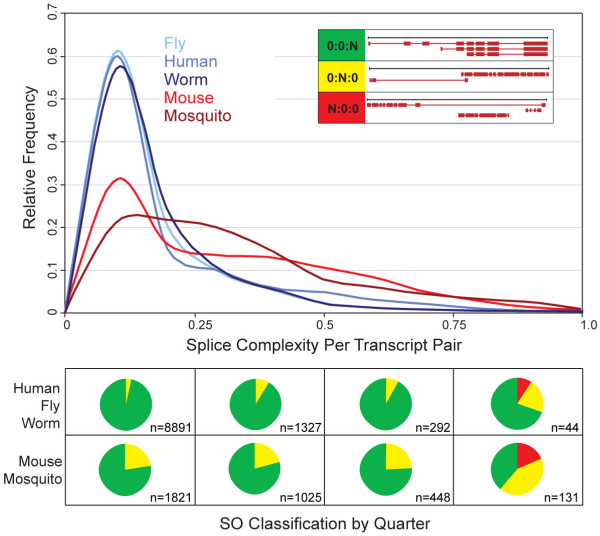
**Genome-wide Splice Complexities and SO classifications of alternatively spliced annotations**. The upper panel shows the frequency distribution of Splice Complexities per transcript-pair. The *x*-axis: Splice Complexity; the *y*-axis: relative frequency. The annotations were then broken into four bins based upon their Splice Complexities: 0.0–0.25, >0.25–0.50, > 0.5–0.75, >0.75–1.0. The contents of each bin were then classified using the SO schema, with the boxes in lower part of the Figure corresponded to the quadrants of the graph above. Each pie chart shows the relative frequency of three different SO-classes for the annotations in that bin: green:0:0:N genes, yellow:0:N:0 genes, red:N:0:0 genes (The insert in top panel provides pictorial summary of the typical splicing patterns associated with these SO classes). The numbers associated with each pie chart represent the total number of annotations in that bin. Pie charts shown in the top-half of the lower panel give the combined breakdown for *H. sapiens*, *D. melanogaster *and *C. elegans *annotations; the bottom-half shows data for the combined *M. musculus *and *A. gambiae *annotations.

The top panel of Figure 5 shows the distribution of Splice Complexities for the current release of each genome in our study. In order to better characterize these distributions, we also broke each genome's alternatively spliced annotations into 4 bins based upon their Splice Complexities and then categorized their contents using the SO classification system (Figure 5, bottom panel). Regardless of genome, annotations with low splice complexities per transcript-pair tend to fall disproportionately into the 0:0:N class, and as Splice Complexity increases there is a concomitant enrichment for problematic annotations that belong to the N:0:0, and 0:N:0 classes. For, example, overall 8% of genes with Splice Complexities between 0.00 and 0.25 fall into the 0:N:0 class, whereas 31% of genes with Splice Complexities greater than 0.75 fall into the 0:N:0 class. Thus, alternatively spliced genes with high Splice Complexities tend to fall into classes that should be prioritized for manual review according to the Sequence Ontology classification system [2].

Interestingly, the *H. sapiens*, *D. melanogaster *and *C. elegans *alternatively spliced annotations all have very similar distributions of Splice Complexity, whereas the *M. musculus *and *A. gambiae *genomes are biased towards higher frequencies of splice- complex annotations (Figure 5, upper panel). The SO based classifications shown in the lower panel of Figure 5 suggest an explanation for these differences. Relative to the other three genomes, *M. musculus *and *A. gambiae *annotations tend to have higher Splice Complexities because they contain more annotations that belong to problematic SO classes. Moreover, the enrichment of these problematic classes grows steadily more pronounced as their Splice Complexity increases (Figure 5, lower panel). These results once again illustrate the utility of our measures for annotation management and meta-analysis and how they complement the SO schema – providing a global overview of an entire genome's alt-spliced genes and allowing the direct comparisons between genomes to reveal an excess of problematic – likely incorrect – annotations in mouse and mosquito genomes that should be subjected to manual review.

Table 4 lists the most Splice Complex annotations from each genome. As might be expected DSCAM [24] has the highest Splice Complexity of any annotation in the *D. melanogaster *genome. This gene is predicted to produce over 32,000 different transcripts, 59 of which are annotated to date. Note however, that even though the Splice complexity of the DSCAM gene is high (149), its average Splice Complexity per transcript pair (0.084) is the lowest of any of the genes in TABLE 4. This indicates that even though DSCAM has many annotated transcripts, on average they are quite similar to one another. Note too that the *M. musculus *and *C. elegans *genes both belong to SO classes indicative of problematic annotations. These results suggest that splice complexity per transcript-pair could be used to help distinguish likely mis-annotated genes from correctly annotated genes, which are simply very complex. Prioritization schemes employing all three measures – total Splice Complexity, Splice Complexity/transcript-pair and SO classification – would likely prove most effective, with genes having high Splice Complexities/transcript-pair and classified into SO class other than 0:0:N heading the list for manual review. Additional File 2 provides a list of such genes compiled from the releases included in our analyses.

**Table 4:** Most complex alternatively spliced annotations.

Organism	Gene	SO Class	Transcript Count	Splice Complexity
*C. elegans*	unc-43 – UNCoordinated family member	2:0:23	25	101 (0.311)
*D. melanogaster*	Dscam – Down syndrome cell adhesion molecule	0:0:59	60	149 (0.084)
*A. gambiae*	GPRGR9	0:0:9	10	34 (0.762)
*M. musculus*	LOC628147 similar to zinc finger protein 709	17:2:24	44	707 (0.747)
*H. sapiens*	CREM – cAMP responsive element modulator	0:0:20	21	53 (0.253)

Columns: organism, gene name, SO classification code (see Figure 4 for key), number of transcripts, Splice Complexity (Splice Complexity per transcript-pair in parenthesis).

### Conservation of Alternative Splicing

We also investigated how often alternative splicing was a trait shared among orthologous loci. EST-based analyses have shown that alternative splicing tends to be conserved even over relatively large phylogenetic distances [25]. We examined to what extent the current crop of annotations capture this fact. We found that alternatively spliced orthologous pairs occur more frequently than would be expected by chance alone. The Human-mouse ODDS RATIO is 1.39; P < 0.001. The *melanogaster*-*gambiae *ODDS RATIO is 1.49; P < 0.001. We also found a statistically significant correlation in the Splice Complexities of these orthologous pairs. The Spearman correlation coefficient [26] of the human-mouse alternatively spliced pairs is 0.36; P < 0.001. It was 0.16; P < 0.001 for *melanogaste*r-*gambiae *alternatively-spliced orthologous pairs. TABLE 5 gives human-mouse, and *melanogaster-gambiae *pairs (as judged by reciprocal best hits) with the greatest differences in Splice Complexity. These facts suggest that the current crop of annotations has only begun to capture the repertory of alternatively spliced transcripts in each genome. The ability to identify pairs of orthologous genes with very different Splice Complexities provides a means to follow up on this hypothesis – further analysis of the member of the pair with the lower Splice Complexity may reveal additional transcripts, not yet annotated, or in cases where there are no missing transcripts, functional differences between the two orthologs.

**Table 5:** Orthologous gene annotations with greatest difference in splice complexity.

Organism	Gene	SO Class	Transcript Count	Splice Complexity
*D. melanogaster*	Dscam	0:0:59	60	148.80 (0.08)
*A. gambiae*	ENSANGG00000015725	0:0:1	2	0.48 (0.48)

*M. musculus*	Sorbs2	0:1:31	33	168.63 (0.32)
*H. sapiens*	Sorbs2	0:0:1	2	0.23 (0.23)

Columns: organism, gene name, SO classification code (see Figure 4 for key), number of transcripts, Splice Complexity (Splice Complexity per transcript-pair in parenthesis). The *D. melanogaster *– *A. gambiae *pair is shown in the top half of the table, the *M. musculus *– *H. sapiens *below. Orthologous genes were identified using reciprocal best hit BLASTP searches.

## Discussion

We have used a variety of new approaches to investigate the annotations of five large eukaryotic genomes, four of them across multiple releases. Our meta-analyses provide novel, global perspectives on the contents of more than 500,000 annotations and their evolution over a period of several years. These analyses have brought to light previously unknown differences and unexpected similarities between their annotations, and allowed us to tease apart differences due in annotation practice from underlying biology. We have also shown how analyses combining Splice Complexity and the Sequence Ontology's classification system can be used to identify and prioritize likely mis-annotated genes for manual review.

Our analyses of Annotation Turnover show that the *H. sapiens *and *M. musculus *annotations are characterized by very high rates of turnover. The major cause of turnover in both genomes appears to be due to incremental changes in the NCBI's annotation protocols, especially as regards pseudogene identification [27]. Since 2003, far fewer annotations have been deleted from either vertebrate genome; and gene addition has been the dominant trend, some of these being resurrected from the earlier releases. This is especially true for mouse, wherein gene numbers rose by 17% between releases 34 and 35. Once again the cause appears to be changing annotation methodologies. Between these two releases the NCBI's gene prediction program, Gnomon, was altered to use a new repeat masking program and to incorporate protein alignments to the genome. This resulted in an increase in gene models in Build 35 compared to Build 34. [27]. For both vertebrate genomes, turnover of Refseq [22] NM and NR annotations has been much lower (Figure 3); these form a stable core amid a continuous flux of more ephemeral annotations.

The high turnover rates characteristic of the human and mouse genomes stand in stark contrast to the more static *D. melanogaster *and *C. elegans *genomes. Almost 99% *of D. melanogaster *annotations present in the omnibus 3.2 release [7], are still present in some form today. *C. elegans *gene numbers are also quite stable, with rates of gene addition and deletion almost balanced – 90% of annotations present in 2003 were still present in 2007 (WS176) and *vice versa*. The stability of gene numbers in both organisms is certainly not due to neglect. Genome-wide searches for new protein coding genes followed by PCR-verification have been undertaken in both animals [28,29].

We used Annotation Edit Distance (Figure 2) to measure active curation independently of annotation turnover. Whereas the *D. melanogaster *annotations are undergoing little revision, the *C. elegans, H. sapiens and Mus musculus *annotations have undergone significant revision with each release. 58% of *C elegans *annotations and 55% of human annotations for example, have been altered since 2003; by comparison only 6% of *D. melanogaster *annotations have been altered during this time. These results show how Annotation Edit Distance can be used to assess the intensity of annotation curation efforts among different databases.

Our analyses of alternatively spliced genes indicate that these are incompletely annotated in every genome in our collection. Despite the fact that alternate splicing is a trait frequently shared among orthologous genes [25,30-32], this trend is poorly captured by the current crop of annotations. For example, estimates based on EST data suggest that around 50% of *D. melanogaster *and *A. gambiae *alternative exons are conserved [25]. At time of writing, however, only 6.4% of *melanogaster-gambiae *orthologous genes are alternatively spliced in both genomes. Likewise, only 2.6% of orthologous human-mouse annotations are alternatively spliced in both genomes, considerably less than the published estimate of 40% based upon EST analyses [30,31]. We did, however, detect a weak but statistically significant tendency for *human-mouse *and *melanogaster-gambiae *orthologs to both be alternatively spliced when either member is. There is also a statistically significant correlation in their Splice Complexities. These facts suggest that the current crop of annotations have begun to capture the conserved aspects of alternative splicing, but that much progress remains possible. Certainly, a rigorous review of alternative splicing patterns among orthologous genes could do much to improve the annotation of all four genomes.

Our analyses using the Sequence Ontology classification system revealed genome specific differences in the frequencies of different modes of alternative splicing. *M. musculus *and *A. gambiae*, for example, are highly enriched for genes whose transcripts share no exon borders in common. Our Splice Complexity based analyses complement these findings: Unexpectedly, the human, *C. elegans *and *D. melanogaster *distributions are all very similar to one another despite the vast evolutionary distances separating these genomes (Figure 5, top panel). This may indicate that common selective forces govern the transcriptional complexity of alternative spliced genes. Inconsistent with this hypothesis, however, the *M. musculus *and *A. gambiae *Splice Complexity distributions are skewed towards higher values due to enrichment for genes with unusual modes of alternative splicing. We believe that annotation quality, rather than biology is the likely cause of the skew towards higher Splice Complexities in these two genomes. If this explanation is correct, the mouse and mosquito distributions should converge upon those of the other three genomes as their annotations mature. But whatever the cause – mis-annotation or fundamental differences in biology – their *in-silico *identification is the first step toward review and experimental investigation of these unusual annotations.

## Conclusion

Although the information encoded in genomic DNA provides a foundation for modern medicine, genome sequences in themselves are not very useful. Their value is dependant upon identifying and annotating the genes they contain. Incomplete and incorrect annotations poison every experiment that employs them. In light of these considerations, accurate and complete genome annotation seems a laudable and achievable goal, especially for model organisms. Because the datasets are so large and complex, *in silico *methods for annotation management must necessarily play a major role in this process. In response, we have formulated three new measures for annotation management – Annotation Turnover, Annotation Edit Distance and Splice Complexity – and used them to investigate the annotations of five genomes. Our results show how these measures can be used to better monitor changes to a genome's annotations from release to release; to compare the magnitude of curation efforts among different genome databases; and to identify and prioritize problematic annotations for manual review.

## Methods

### Tracking annotations from release to release

Reciprocal best hits are commonly used to identify orthologous genes, even over large evolutionary distances [10,33]. This approach is also effective for tracking annotations from assembly to assembly, as intra-genome differences are meager in comparison to cross-genome differences. In order to determine the accuracy of this procedure, we used two complementary approaches. First, we searched the first and last release from each genome against themselves. We found that on average, 98.7% of genes were their own reciprocal best hits; this percentage demonstrates that paralogs, repeats and low complexity sequence have little impact on the accuracy of the reciprocal best hits procedure. We used a second procedure to assess the impact of greater release distance on accuracy. To do so, we identified reciprocal best hits between each release and its closest two temporal neighbors, and used these data to populate a graph of reciprocal best hits from release to release for each genome. We then compared the correspondence between reciprocal best hits obtained by traversing this graph from start to end to those obtained from searching the most current release against the earliest release. The trace-based approach recovered a subset (91%) of the reciprocal best hits obtained by the first approach. For *D. melanogaster *and *C. elegans *the percentage was 100% and 98%, respectively. For *H. sapiens *and *M. musculus *it was 85% and 79%. Resurrection of previously deleted genes lowered percentages in the two vertebrate genomes. For these reasons we conclude that simply blasting releases against one another is the preferred means of tracking annotations across releases. All searches were preformed with the following WU-BLAST command: blastn <db> <query> -filter = seg -cpus = 1 -W = 30 -N = -10 -mformat = 2 -B = 1 E = 1e-6 -gspmax = 5 T = 1000 -wink = 30.

### Assembly-induced Changes

Changes to the underlying assembly complicate analyses of annotation change. We therefore sought to segregate changes to annotations resulting solely from curation, from those resulting from changes to the underlying assembly. To do so, we first identified versions of the same annotation in sequential pairs of releases using a reciprocal best hits approach. We then compared the underlying genomic sequences (including a flanking region of 500 bp) for each gene version-pair. If there was any change to the underlying genomic sequence, these annotations were flagged as altered due to assembly change. We found that the impact of assembly changes on existing annotations varied widely from genome to genome and from release to release (Additional File 3). The *D. melanogaster *and *C. elegans *assemblies were the most static; on average only 0.42% of *D. melanogaster *and 0.30% of *C. elegans *genes experienced changes to their underlying DNA sequences from release to release. The *H. sapiens *and *M. musculus *assemblies were more labile. On average 3% of *H. sapiens *and 18% of *M. musculus *annotations underwent assembly induced coordinate changes from release to release. For both vertebrate genomes the vast majority of these occurred between early releases. In *M. musculus*, for example, the underlying genomic sequences of 30% of release 30 (02/2003) annotations had been altered by 32.1 (10/2003), whereas the percentage fell to 15% between releases 34.1 (05/2005) and 35.1 (09/2005) and to only 0.05% between releases 35.1 (09/2005) and 36.1 (05/2006). *H. sapiens *followed a similar trend (averaging around 3% release), with the exception of release 35.1, which had a higher percentage (5%).

### Calculating Annotation Edit Distance

Sensitivity, Specificity, and Accuracy [13] are commonly used to measure gene-finder performance relative to some standard, usually a reference annotation that is well supported by experimental evidence. Sensitivity (SN) is the fraction of the reference feature predicted, whereas Specificity (SP) is the fraction of the prediction overlapping the reference feature. Both measures can be calculated for any feature class, *e.g*. transcripts, exons or introns; and the calculations can be preformed at the nucleotide level, or, if greater stringency is desired, the fraction of the features predicted exactly [23]. SN and SP are often combined into a single measure called Accuracy (AC). Several formulations of accuracy are in use (see [13]). Some of these take true negatives into account; others do not. In practice, it can be difficult to determine the scope of true negatives for genome annotations, as these can be considered as limited to some flanking region around the gene in question, the entire intergenic region or even the rest of the genome. Including true negatives in the accuracy calculation also complicates *inter*-genome comparisons. For example, gene-prediction accuracy will tend to be higher for those genomes with large introns and intergenic regions. For these reasons we have used a simple average, (SN +SP)/2, to measure accuracy.

**Figure 6:**
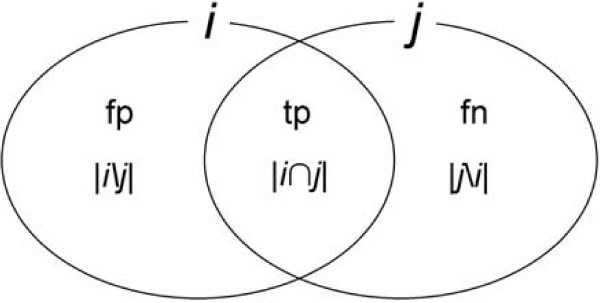
**Components of the SN and SP calculations and their set-theoretic equivalents**. Venn diagram showing the relation of true positives (tp), false negatives (fn) and false positives (fp) to their set-theoretic equivalents. |*i*∩*j*|: number of nucleotides shared in common between annotations *i *and *j*; |*j*\*i*|: nucleotides in *j*, not in *i*; |*i*\*j*|: nucleotides in *i*, not in *j*.

Although SN, SP and AC are normally thought of as measures of agreement between a prediction and a reference annotation, there is no inherent requirement for a reference annotation. The measures can also be used to compare two annotations to one another. Reformulating SN in terms of sets makes this clear (see Figure 6). SN for example is usually given as SN = tp/(tp + fn), where tp is number of true positives and fn false negatives. But SN can also be thought of as the fraction of annotation *i *overlapping annotation *j*. Substituting tp and fn for their set-theoretic equivalents (Figure 6), SN = |*i*∩*j*|/(|*i*∩*j*| + |*j*\*i*|), where |*i*∩*j*| is the number of overlapping nucleotides (tp), and |*j*\*i*| the number of nucleotides in *j *not annotated in *i*, or fn. Since, by definition |*j*| = |*i*∩*j*| + |*j*\*i*|, SN = |*i*∩*j*|/|*j*|, or the fraction of *j *overlapping *i*. Likewise, SP can be thought of as the fraction of *i *overlapping *j*, and Accuracy (AC) as the average of these two fractional overlaps – a bi-directional measure of *Congruency *between two annotations that we denote as *C*. The *incongruence *or distance, *D*, between annotation versions *i *and *j *then becomes *D *= 1-*C*.

**Figure 7:**
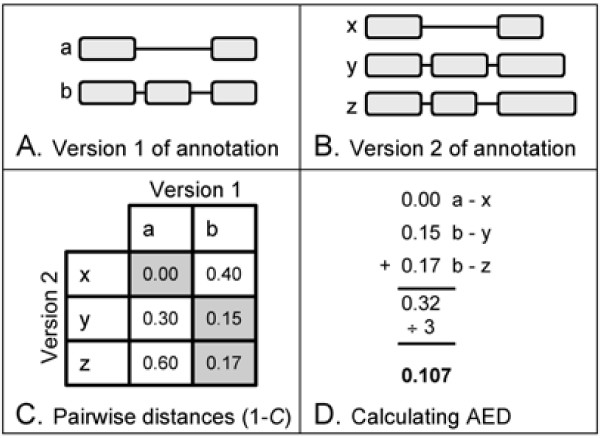
**Calculating the distance between two versions of the same annotation**. Panels A & B: two versions of the same annotation. C. Pairwise distances between transcripts of version 1 and 2. Minimum distances are highlighted; (D) these are summed to give a value for the gene as a whole (0.32) or normalized by the number of transcript-pairs to give an average per transcript-pair (0.107).

So long as both versions of the annotation contain only a single annotated transcript, AED, is easily calculated. Alternative splicing, however, complicates matters somewhat. The problem lies in how best to pair the transcripts of one version of the annotation with those of another. Several different procedures can be envisioned; we have chosen one that will always give the minimal distance. The procedure is shown in Figure 7. First, pairwise incongruencies, or 1-*C*, between each possible pairing of annotation *i*'s transcripts with those of *j *are calculated. Each transcript is then paired with its closest partner from the other annotation. In cases where a transcript has multiple equidistant partners, one of these is chosen randomly. In cases where the two annotations have different numbers of transcripts, two transcripts from one version can share the same partner in the other annotation. The pairwise distances are then summed (Figure 7, panel D). The result is a (minimum) measure of distance between two versions of the gene, which we term Annotation Edit Distance, or AED. This value can also normalized by the number of transcript-pairs to give the average AED/transcript-pair, a number useful for analyses of alternatively spliced annotations. AED is a general measure, not restricted to transcripts. This makes it possible to compute multiple, feature-specific AEDs for purposes of better annotation management. For example, changes to UTRs between releases can be analyzed independently of changes to other features. Moreover, AED is genome independent; this means that the magnitude of release-to-release revisions can be compared across different genomes as we have done in Figure 2.

### Splice Complexity

**Figure 8:**
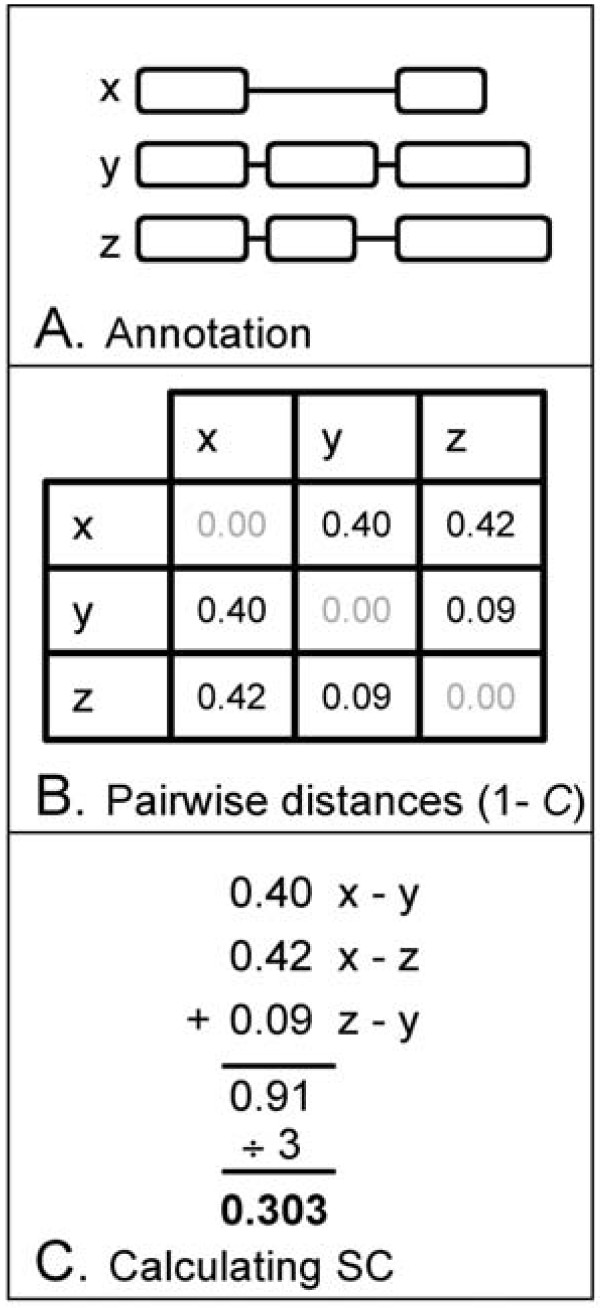
**Calculating Splice Complexity**. Pairwise distances between transcripts of an annotation (A) are shown in (B). Minimum distances between each transcript-pair can be summed (C) to give a value for the gene as a whole (0.91) or normalized by the number of transcript pairs (0.303).

Annotators often speak of a gene as having simple or complex pattern of alternative splicing, but to date complexity has been a term without a precise meaning. In response, we have developed a quantitative measure of annotation complexity that we term Splice Complexity. Splice Complexity is closely related to AED, and is calculated as follows (see Figure 8). First the incongruence, 1-*C*, is calculated for every pairwise combination of an annotation's transcripts. Next, these values are summed. We term the result the gene's Splice Complexity. Splice Complexity can also be normalized by the number of transcript-pairs to give an average complexity per transcript-pair. Splice Complexity is thus quite similar to AED, but whereas AED is used to measure the distance *between *two versions of the same annotation, Splice Complexity can be thought of as an *intra*-*annotation *measure of its complexity. Importantly, Splice Complexity provides a measure of annotation complexity that is independent of sequence similarity. This means that the genome-wide complexity of alternative splicing in different genomes can be compared to one another as we have done in Figure 5. Though we have restricted the analyses reported here to transcript-level comparisons, Splice Complexity can also be used for comparisons of orthologous and paralogous genes – and provides a means to identify pairs of such annotations that have widely differing complexities (TABLE 5). These characteristics make it a useful measure for both annotation quality control and comparative genomics.

### Annotation Turnover

From release to release annotations are added, deleted, split and merged. Because gene numbers only tally the ratio of additions to deletions they give little insight into the process of annotation turnover. An obvious approach to investigating annotation turnover would be to follow gene IDs from release to release, but in practice this proved problematic for some of the earlier releases. Instead, we used a reciprocal best-hits approach to investigate the process of annotation turnover, as this provides a general method not dependent upon ID history data, which is not always available. For each genome's collection of releases we searched the transcripts from the most recent release in our collection against the earlier releases and *vice versa*. If one or more of a gene's alternative transcripts had a reciprocal best hit to a transcript in an earlier release, that gene was considered present in that release, but only so long as all its transcripts' reciprocal best hits were to the same target gene and *vice versa*. We used exactly the same procedure to track annotations in the other direction as well, *i.e*. from the first release for each genome forward through subsequent releases.

### Datasets

*H. sapiens*: GenBank releases 33 (04/2003), 34.1 (10/2003), 34.2 (01/2004), 34.3 (03/2004), 35.1 (08/2004), 36.1 (03/2006), 36.2 (09/2006). *M. musculus*: GenBank releases 30 (02/2003), 32.1 (10/2003), 33.1 (09/2004), 34.1 (05/2005), 35.1 (09/2005), 36.1 (05/2006). *D. melanogaster*: FlyBase releases 3.1 (10/2004), 4.2 (09/2005), 4.3 (03/2006), 5.1 (12/2006). *C. elegans*: WormBase releases WS100 (05/2003), WS130 (09/2004), WS150 (11/2005), WS160 (07/2006), WS 176 (06/2007). *A. gambiae *GenBank release (downloaded 08/2007). The *H sapiens*, *M. musculus *and *A. gambiae *releases were downloaded from . *The D. melanogaster *releases were downloaded from , and *the C. elegans *releases from .

### Software

GenBank releases were converted to Chaos-XML prior to processing using cx_genbank2chaos.pl . Older gff3 releases from WormBase were brought forward to current gff3 specifications  using the scripts ws100_forward, ws130_forward, and ws150_foward. Bulk gff3 files for *D. melanogaster *and *C. elegans *chromosomes were split into individual annotations along with their accompanying nucleotide sequence using the cgl-gff3 library . Annotation Edit Distances and Splice Complexities were calculated at the nucleotide level using the scripts splice_distance_nucleo and splice_complexity_nucelo respectively. All code is available at . After download the bundle should be uncompressed. A README details requirements and the installation procedure.

## Authors' contributions

KE and MY conceived the study, carried out analyses, wrote software, and wrote paper. BM and CH carried out experiments and wrote software for the analyses.

## Supplementary Material

Additional file 1 **Release Dates, Gene and Transcript Counts**. Columns: release name, release date, gene count, transcript count and number of genes annotated with multiple transcripts. Data shown for each release analyzed in this study for *H. sapiens*, *M. musculus*, *D. melanogaster, A. gambiae *and *C. elegans*. The number in the 'Genes' column represents the number of records tagged as a gene in either the GenBank or GFF3 files for that organism and release. For the GFF3 files this is limited to protein-coding genes, as variability in early GFF3 formats precluded the inclusion of non-coding RNA genes. The number in parenthesis is the number of genes used in our analyses. There are a variety of reasons for the differences between the raw gene count and the number of genes that we analyzed. In general if there were annotations to support, or if we could infer, a valid gene model from the contents of the gff3 or GenBank file, with at least one transcript and exon then we analyzed the record. GenBank records for human and mouse annotate some pseudogenes with transcripts (but not all) and we have included those genes with transcripts in our analyses. Finally, there are some records for which – due to incomplete or corrupt annotations – a valid gene model cannot be inferred. We have excluded them from our analyses. The numbers in the 'Transcripts' column represent a count of records in GenBank files that have a transcript_id tag, and in fly and worm GFF3 files, records that have a type field with mRNA. The values in the 'Alt. Spliced Genes' column represent the number of genes included in our analyses which had more than one transcript associated with them. Click here for file

Additional file 2 **Genes with annotations that may need review**. Top ten problematic genes from the most recent release for each genome in our dataset. Genes were prioritized first on the basis of having SO-classifications indicative of problems, and second on Splice Complexity. These criteria identified only seven genes in *D. melanogaster* Click here for file

Additional file 3 **Number of version pairs with assembly induced coordinate changes**. The number of genes for each release pair that were excluded from Annotation Edit Distance calculations due to sequence changes within the gene region. Click here for file
