# Supplementary material for: Quantitative measures for the management and comparison of annotated genomes
Source: BMC Bioinformatics. 2009 Feb 23;10:67. doi: 10.1186/1471-2105-10-67 (PMC2653490; doi:10.1186/1471-2105-10-67)
Supplement: Additional file 1 — Release Dates, Gene and Transcript Counts. Columns: release name, release date, gene count, transcript count and number of genes annotated with multiple transcripts. Data shown for each release analyzed in this study for H. sapiens, M. musculus, D. melanogaster, A. gambiae and C. elegans. The number in the 'Genes' column represents the number of records tagged as a gene in either the GenBank or GFF3 files for that organism and release. For the GFF3 files this is limited to protein-coding genes, as variability in early GFF3 formats precluded the inclusion of non-coding RNA genes. The number in parenthesis is the number of genes used in our analyses. There are a variety of reasons for the differences between the raw gene count and the number of genes that we analyzed. In general if there were annotations to support, or if we could infer, a valid gene model from the contents of the gff3 or GenBank file, with at least one transcript and exon then we analyzed the record. GenBank records for human and mouse annotate some pseudogenes with transcripts (but not all) and we have included those genes with transcripts in our analyses. Finally, there are some records for which – due to incomplete or corrupt annotations – a valid gene model cannot be inferred. We have excluded them from our analyses. The numbers in the 'Transcripts' column represent a count of records in GenBank files that have a transcript_id tag, and in fly and worm GFF3 files, records that have a type field with mRNA. The values in the 'Alt. Spliced Genes' column represent the number of genes included in our analyses which had more than one transcript associated with them. [file 1471-2105-10-67-S1.pdf]

Additional Table 1

Release Dates, Gene and Transcript Counts

Homo sapiens

Release	Release Date	Genes	Transcripts	Alt. Spliced Genes
33	Apr-03	36,117 (33,000)	37,854	1,540
34.1	Oct-03	26,123 (22,956)	26,213	1,841
34.2	Jan-04	27,490 (23,163)	26,868	2,120
34.3	Mar-04	28,402 (24,045)	28,139	2,375
35.1	Aug-04	26,849 (23,245)	27,552	2,498
36.1	Mar-06	29,104 (21,812)	32,366	3,816
36.2	Sep-06	28,755 (23,342)	31,118	3,614

Mus musculus

Release	Release Date	Genes	Transcripts	Alt. Spliced Genes
30	Feb-03	36,443 (34,377)	36,112	138
32.1	Oct-03	28,977 (26,006)	26,405	301
33.1	Sep-04	27,238 (24,933)	25,540	432
34.1	May-05	27,513 (25,200)	25,975	525
35.1	Sep-05	32,287 (31,524)	37,782	2,278
36.1	May-06	31,805 (31,037)	37,280	2,254

Drosophila melanogaster

Release	Release Date	Genes	Transcripts	Alt. Spliced Genes
r3.2	Oct-04	13,472 (13,472)	19,302	2,906
r4.2	Sep-05	14,380 (14,330)	19,178	3,332
r4.3	Mar-06	14,449 (14,398)	19,376	3,396
r5.1	Dec-06	14,601 (14,512)	19,781	3,173

Anopheles gambiae

Release	Download Date	Genes	Transcripts	Alt. Spliced Genes
current	May-06	12,472 (12,470)	13,840	1,184

Caenorhabditis elegans

Release	Release Date	Genes	Transcripts	Alt. Spliced Genes
WS100	May-03	19,559 (19,404)	21,565	1,443
WS130	Sep-04	21,379 (19,601)	24,298	2,530
WS150	Nov-05	21,879 (20,062)	26,198	3,600
WS160	Jul-06	22,066 (20,080)	27,015	3,995
WS176	Jun-07	20,061 (20,061)	26,867	3,858
