# Supplementary material for: Quantitative measures for the management and comparison of annotated genomes
Source: BMC Bioinformatics. 2009 Feb 23;10:67. doi: 10.1186/1471-2105-10-67 (PMC2653490; doi:10.1186/1471-2105-10-67)
Supplement: Additional file 2 — Genes with annotations that may need review. Top ten problematic genes from the most recent release for each genome in our dataset. Genes were prioritized first on the basis of having SO-classifications indicative of problems, and second on Splice Complexity. These criteria identified only seven genes in D. melanogaster [file 1471-2105-10-67-S2.pdf]

Supplemental Table 2

Genes With Annotations That May Need Review

	Gene	SO Code	SC
H. sapiens	NBPF1	13:0:2	73
	NBPF10	12:2:80	2367
	C3orf41	2:0:2	8
	ZNF365	1:0:2	4
	WWP2	1:0:1	2
	SVEP1	1:0:4	9
	PDE4DIP	1:0:3	7
	KALRN	1:0:1	2
	INPP5F	1:0:1	2
	FLJ31033	1:0:4	11
M. musculus	Phf20l1	6:0:7	60
	Whsc1l1	3:0:5	25
	Rgs11	3:3:8	54
	Phip	3:2:5	27
	C330002I19Rik	3:0:10	37
	Srgap2	2:0:10	31
	Kalrn	2:0:5	20
	Flnb	2:2:0	5
	Clasp1	2:0:13	34
	C030046E11Rik	2:0:2	7
A. gambiae	ENSANGG00000006332	4:0:0	6
	ENSANGG00000017120	2:0:0	3
	ENSANGG00000019640	1:1:1	5
	ENSANGG00000018885	1:0:0	1
	ENSANGG00000018868	1:0:0	1
	ENSANGG00000018756	1:0:0	1
	ENSANGG00000018693	1:0:0	1
	ENSANGG00000017543	1:0:2	5
	ENSANGG00000017049	1:0:1	2
	ENSANGG00000016139	1:0:0	1
C. elegans	WBGene00019237	4:0:2	7
	WBGene00018101	3:1:1	8
	WBGene00009180	3:0:3	12
	WBGene00008555	3:0:0	3
	WBGene00006975	3:0:1	5
	WBGene00006805	3:0:3	10
	WBGene00020784	2:0:1	3
	WBGene00017098	2:0:2	6
	WBGene00016201	2:0:3	7
	WBGene00015887	2:2:1	10
D. melanogaster	FBgn0013576	4:0:10	32
	FBgn0001624	4:0:5	25
	FBgn0052158	3:0:2	8
	FBgn0013988	3:0:3	12
	FBgn0052425	1:0:2	2
	FBgn0036478	1:0:1	2
	FBgn0026193	1:0:13	46
