# Supplementary material for: Quantitative measures for the management and comparison of annotated genomes
Source: BMC Bioinformatics. 2009 Feb 23;10:67. doi: 10.1186/1471-2105-10-67 (PMC2653490; doi:10.1186/1471-2105-10-67)
Supplement: Additional file 3 — Number of version pairs with assembly induced coordinate changes. The number of genes for each release pair that were excluded from Annotation Edit Distance calculations due to sequence changes within the gene region. [file 1471-2105-10-67-S3.pdf]

Additional Table 3

Number of version-pairs with assembly induced coordinate changes

Organism	Release	# Sequence Pairs
H. sapiens	33-34.1	1,104
	34.1-34.2	619
	34.2-34.3	484
	34.3-35.1	1,281
	35.1-36.1	500
	36.1-36.2	198
M. musculus	30-32.1	10,133
	32.1-33.1	7,642
	33.1-34.1	4,505
	34.1-35.1	3,601
	35.1-36.1	16
D. melanogaster	r3.2-r4.2	133
	r4.2-r4.3	18
	r4.3-r5.1	23
C. elegans	WS100-WS130	151
	WS130-WS150	30
	WS150-WS160	21
	WS160-WS176	36
